# The most effective sexual function and dysfunction interventions in individuals with multiple sclerosis: A systematic review and meta-analysis

**DOI:** 10.18502/ijrm.v20i4.10897

**Published:** 2022-05-23

**Authors:** Bahare Afshar, Leila Amini, Maryam Hasani, Shayesteh Jahanfar, Seyed Massood Nabavi

**Affiliations:** ^1^Student Research Committee, School of Nursing and Midwifery, Iran University of Medical Sciences, Tehran, Iran.; ^2^Nursing Care Research Center (NCRC), Department of Midwifery, School of Nursing and Midwifery, Iran University of Medical Sciences, Tehran, Iran.; ^3^Department of Midwifery, School of Nursing and Midwifery, Lorestan University of Medical Sciences, Khoramabad, Iran.; ^4^MPH Program, Department of Public Health and Community Medicine, Tufts University School of Medicine, Boston, USA.; ^5^Department of Regenerative Biomedicine, Cell Science Research Center, Royan Institute for Stem Cell Biology and Technology, ACECR, Tehran, Iran.; ^6^Department of Brain and Cognitive Sciences, Cell Science Research Center, Royan Institute for Stem Cell, Tehran, Iran.

**Keywords:** Clinical trial, Multiple sclerosis, Sexual dysfunction, Systematic review.

## Abstract

**Background:**

Sexual dysfunction has many factors in multiple sclerosis, but there is no reliable treatment for this challenge.

**Objective:**

Determining effective sexual function or dysfunction interventions in individuals with multiple sclerosis.

**Materials and Methods:**

To find the relevant published interventional studies that at least had an English abstract or in Persian, we searched International Statistical Institute, PubMed, Scopus, Cochrane, Medline, PsycINFO, EMBASE, CINAHL, and Google Scholar from January 1990 to June 2021. The results were analyzed using RevMan 5.3 software. The p < 0.05 was considered significant.

**Results:**

Out of 568 articles, 41 were included after deleting the duplicate and irrelevant articles. Studies were divided into 2 groups of sexual function (n = 27) and dysfunction (n = 14). Interventions in each category have 4 subgroups: psychoeducational, exercise and rehabilitation, and medical and multi-type interventions. For improving sexual function, more than half of psychoeducational interventions showed a significant improvement after interventions (p = 0.0003). In sexual dysfunction studies, most of the interventions (n = 13/14) had improved at least one subscale of sexual dysfunction. Medical interventions were effective on men's sexual dysfunction, and psychoeducational interventions had been more effective in women's sexual dysfunction.

**Conclusion:**

Psychoeducational and medical interventions are the commonest effective interventions. The psychoeducational studies conducted specifically on women had a positive impact, and only 4 articles with medical interventions were specifically targeted at men, which had a positive effect.

## 1. Introduction

Multiple sclerosis (MS) is a central nervous system disease with physical, emotional, and cognitive functioning changes which causes many problems in the daily living activities performance (1). For treating MS and controlling the symptoms, experts were focused on pharmaceutical interventions and immune-modulating drugs (2).

Sexual dysfunction occurs due to many factors such as neurological lesions of MS, psychological factors, side effects of meditations, fatigue, muscular weakness, pain, bladder or bowel incontinency or other disease manifestations (3, 4). Neurogenic sexual dysfunction often severely affects the quality of life. Therefore health care professionals should pay attention to this aspect of the health of MS individuals (5, 6). Sexual disorders in MS individuals can be caused by various mechanisms (7). Moreover, secondary sexual dysfunction can occur because of other MS symptoms (8, 9). Individuals with MS in both genders commonly face sexual dysfunction (10). However, women have reported more dysfunction in sexual life than men (11, 12). Men may experience difficulty achieving and/or maintaining an erection and decreased recurrence of ejaculation (13-16). There have been many interventions to improve the symptoms and relieve the pepole with MS (17).

To determine the effective interventions on sexual function or dysfunction in MS people, this systematic review and meta-analysis was designed.

## 2. Materials and Methods

This systematic review was carried out following PRISMA guidelines (18). A systematic search was performed over electronic databases including the International Statistical Institute, PubMed, Scopus, Cochrane, Medline, PsycINFO, EMBASE, CINAHL, and Google Scholar. The search was performed on each database using the keywords listed in table I in titles/abstracts.

All kinds of interventional published studies in English or Persian related to the sexual function or dysfunction in people with MS were entered. The search was performed between January 1990 and June 2021. Data extraction was conducted by 2 authors independently. First, articles were imported to EndnoteX7, and duplicate articles were removed. The remaining studies were included in the full paper screening. While reviewing the titles and abstracts, studies that did not match the review objectively and those that could not provide adequate information were removed. Each article was scored based on a checklist and the proportion of each item was calculated. To determine the article's level, articles were divided into 3 categories of poor (0-12), medium (13-25), and good quality (26-37).

In addition, the risk of bias performed using the Review Manager 5.3 software using 6 items, including; random sequence generation (selection bias), allocation concealment (selection bias), blinding of participants and personnel (performance bias), blinding of outcome assessment (detection bias), incomplete outcome data (attrition bias), selective reporting (reporting bias). We extracted the mean score of sexual function or dysfunction before and after the interventions, or mean difference. All the included studies in the current systematic review were organized in 2 separate tables to evaluate the effect of interventions on sexual function/ dysfunction. The necessary data were excluded and written. For meta-analysis of the data, we need articles that reported the mean difference and standard deviation and used a similar questionnaire. The data were analyzed using the RevMan 5.3 software (19). Further heterogeneity was explored using the Chi-square test at the 5% significance level (p < 0.05).

**Table 1:** Search term


**Block 1: **"Multiple sclerosis" OR MS
**Block 2:** Sex* OR intimacy OR intercourse OR coitus OR “sexual relation” OR coition OR “sexual behavior*” OR “sexual function” OR “sexual dysfunction” OR “sexual satisfaction” OR “sexual problems” OR “sexual difficulties” OR “sexual desire” OR “sexual arousal” OR “Orgasm” OR “erect*” OR “lubricant” OR “sexual intercourse” OR “sexual activity” OR “sexual practice*” OR “sexual preference*” OR “vaginal sex” OR “anal sex” OR “oral sex” OR “sexual life” OR “sex life” OR “sexual experience*” OR “sexual Intercourse” OR “intercourse behavior*” OR “intercourse activity” OR “intercourse practice*” OR “intercourse preference*” OR “vaginal intercourse” OR “anal intercourse” OR “intercourse experience*” OR Coitus OR “coital behavior*” OR “coital activity” OR “coital practice*” OR “coital preference*” OR “vaginal coitus” OR “anal coitus” OR “coital experience*” OR coital OR frequency OR “sexual frequency” OR “intercourse frequency” OR “coital frequency” OR “frequency change*” OR Prevalence OR “number of times” OR Change* OR increase OR decrease OR decline OR modify OR reduce* OR reduction OR less* OR more OR variation OR mutation OR ratio OR “sexual assertiveness” OR “hedonistic behavior” OR “sexual interest” OR “sexual motivation” OR quality of life
**Block 3:** “Randomized control trial” OR RCT OR “randomized trial” OR “intervention*” OR improvement OR management

## 3. Results

**Figure 1:**
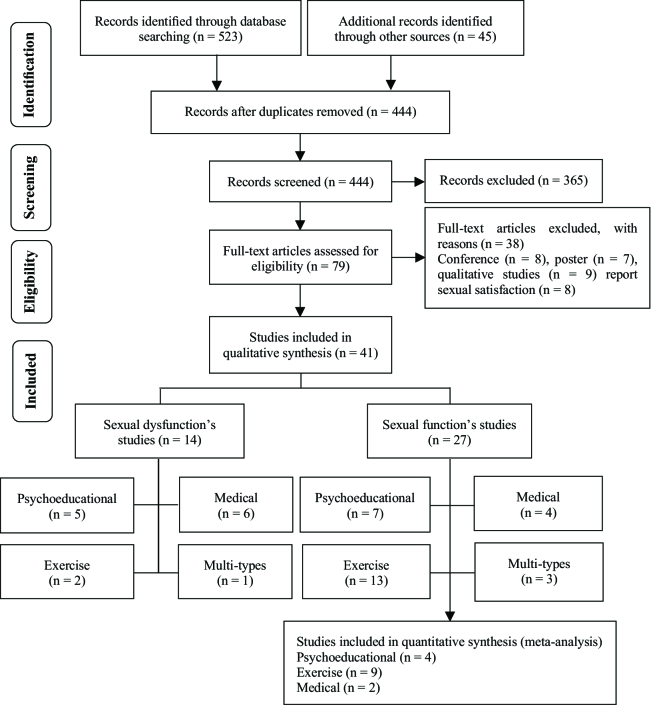
PRISMA flow diagram.

568 articles were imported to EndnoteX7, and 124 duplicate articles were removed. The remaining studies (n = 444) were included in the full paper screening. While reviewing the titles and abstracts, 365 studies did not match the review objective, 38 studies could not provide adequate information. Thus, 41 studies remained in this systematic review (Figure 1).

Generally, the studies were divided into 2 groups. The first category of studies (n = 27) examined “sexual function” in a population with MS without screening for sexual dysfunction disorder.

The second group (n = 14) considered sexual dysfunction in people with MS who had sexual problems. Studies on sexual dysfunction had primary inclusion criteria; participants were required to demonstrate symptoms of sexual dysfunction based on scales or people's statements.

Thus the results were interpreted based on evaluating interventions to improve sexual function or treatment of sexual dysfunction.

### Improvement of sexual function

#### Characteristics of studies

Among 27 articles from 2001-2020, which reported sexual function's changes during interventional studies, 15 were randomized control trials, 9 were quasi-experimental, an article in each cohort, a non-randomized control trial, and clinical trial designs. In 23 articles, sexual function was extracted from MS quality of life-54 (MSQOL-54) questionnaire subscales, and only 4 articles specifically examined sexual function with female sexual function index (FSFI), which is a specific questionnaire of sexual. From a total sample size of (n = 27 studies) 1248 participants, 733 patients were female, and 214 of them were male. In 4 articles, the gender of participants was not noted (n = 301). The range of age in participants was varied from 29.80-48.20, and the range of expanded disability status scale was 1.9-6.9 in reported studies.

**Figure 2:**
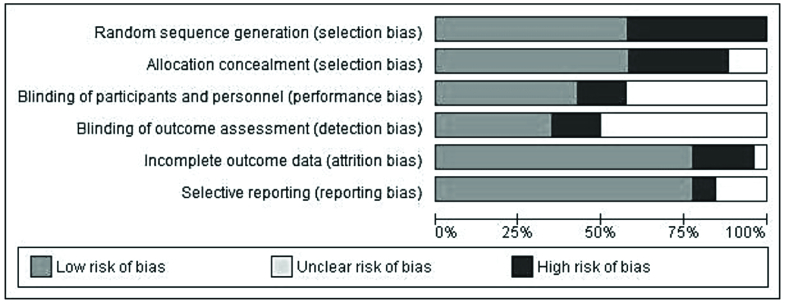
The risk of bias in sexual function studies.

All the interventions in this session were divided into 4 classifications of psychoeducational interventions (n = 7), exercise and rehabilitation interventions (n = 13), medical interventions (n = 4), and multi-types-interventions (n = 3) (used more than one type of interventions). 22 (n = 6) articles were classified as medium, and 78% (n = 21) were classified as studies with good quality. Also, 15 of the 37 items on the CONSORT checklist were reported in 100% of the studies. The risk of bias was determined and shown in figure 2.

#### Psychoeducational interventions (n = 7)

Duration of Educational interventions was varied between 2-8 sessions of 40-50 min in about 12 wk; also, the psychological interventions were held between 6-20 sessions of 60-90 min, once or twice a week (19-25). These interventions include; educational intervention: based on the extracted educational needs of the participants, self-care program based on different methods, including the Orem model and training program. Psychological intervention: emotionally focused therapy, mindfulness-based stress reduction program, happiness therapy based on seligman's positive approach, and short-term dynamic psychotherapy. Finally, 5 articles could have improved sexual function in MSQOL-54 subscales (20-22) and subscales of FSFI (19, 23). Out of the 7 studies in the systematic review of this classification, 4 reported the mean and SD, so they entered meta-analysis, and used sensitivity analysis to decrease heterogeneity. A relatively low heterogeneity was identified among studies.

According to table II, there is no significant difference between the experimental and control groups in the pre-test (p = 0.33). Still there is a significant effect between the 2 groups (p = 0.0003) (Table III). Thus, psychoeducational interventions can improve sexual function.

#### Exercise and rehabilitation interventions (n = 13)

These interventions were designed from 8-36 sessions of 30-90 min during 8-10 wk (26-38). The exercises were included; aerobic, yoga, treadmill, aquatic exercise, relaxation, mindfulness training, Tai Chi Chuan exercise, resistance, and proprioceptive neuromuscular facilitation. Overall, just 2 studies had a positive effect on sexual function, one of them extracted from MSQOL-54 subscales, it was about 3 supervised training sessions per wk for 10 wk, apart from 5 sessions during weeks 5 and 6, which involved land-based weight training, the program was conducted in water (35). The other study was assessed the impact of aquatic exercising, which interventions had significant and positive effects on the overall score of sexual function (p < 0.001) and all the subscales of FSFI (38). 9 studies that used the MSQOL-54 questionnaire and reported the mean and standard deviation were included meta-analysis; we used random effects and sensitivity analysis to decrease heterogeneity. The result of the Chi^2^ test for heterogeneity showed that there was considerable homogeneity among the included studies.

The sexual function means difference was not significant between the 2 groups before (p = 0.34) and after (p = 0.86) the intervention in exercise and rehabilitation classification (Table III). Thus, these interventions are likely to have little effect on sexual function.

#### Medical interventions (n = 4)

All the medicines used in this category were MS therapeutic drugs or supplements. MS therapeutic drugs: Using Interferon beta-1a or Interferon beta-1b during 2 year of follow-up (39). 2. 30 μg Avonex once/wk via intramuscular injection, or 44 μg Rebif 3 times/wk via subcutaneous injection, or 0.25 mg Betaferon every other day via subcutaneous injection, in comparison with each other (40). They both couldn't improve sexual function anymore.

Supplements drugs: Receiving 2 doses of 2,000 vitamin D pills for 12 wk improved orgasm, satisfaction, pain, and overall FSFI score (41). Or 2.5 mg of folic acid daily for 2 months and 3 doses of 1 mg vitamin B12 had a positive and significant effect on sexual function in both intervention and placebo groups. Still, there was no significant difference between the 2 groups after the intervention (42). Just 2 studies that used MSQOL-54 and reported mean difference and SD entered this session of meta-analysis. In the pre-test session, the I^2^ was estimated at 26%, which means we can be ignored heterogeneity, but there is significant heterogeneity among studies in the post-test (Table IV).

According to a p-value of pre and post-treatment, this kind of medical intervention could not improve sexual function.

#### Multi-types-interventions (n = 3)

Interventions in 3 articles were designed based on more than one type of method; one of them was based on exercise and psychoeducational methods, and it didn't have any positive effect on sexual function (43). 2 others were conducted on sun exposure and vitamin D supplementation, meditation, exercise, and stress reduction and showed a positive effect on sexual function (44).

The second one was performed on 1 methylprednisolone and physiotherapy, education, bladder management techniques, self-management, exercising during steroid therapy (45). Psychoeducational interventions are likely to be more acceptable to promote sexual function. Even if they are not specifically designed to promote sexual issues, they can promote sexual function.

### Treatment of sexual dysfunction

14 articles were entered in the second category that 7 studies were randomized control trials, 7 were a clinical trial, and the other was quasi-experimental. 10 articles were conducted with female participants (n = 433) and 4 articles were conducted on men (n = 532) so the total sample size in this section of a systematic review is 965. The age of participants ranged from 34-47. Sexual dysfunction was assessed by FSFI, MS intimacy and sexuality questionnaire-19, sexual function questionnaire, and International Index of Erectile Function Questionnaires (46, 47). All interventions were divided into 4 categories: Psychoeducational interventions (n = 5), exercise and rehabilitation interventions (n = 2), medical interventions (n = 6), and a multi-type intervention study.

#### Risk of bias and quality assessment

**Figure 3:**
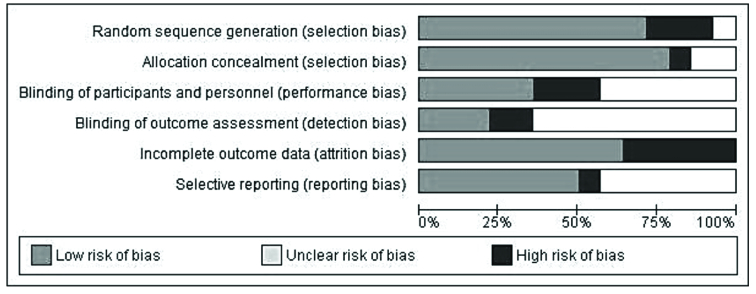
The risk of bias in sexual dysfunction studies.

100% (n = 14) of articles were classified as studies with good quality, and there is no article in 2 other classifications. Also, 21 of the 37 items on the Consort checklist were reported in 100% of the studies. Figure 3 shows the risk of bias in sexual dysfunction studies.

#### Psychoeducational interventions (n = 5)

The number of sessions varied from one to 12, and each session took from 20-90 min, during 2-12 wk (46, 48-51). This category includes written materials on sexual dysfunction education in participants with MS with the introduction of additional sources VS written materials as well as 3 sexual counseling sessions, sexual counseling mostly based on PLISSIT & Ex-PLISSIT models, emotionally focused therapy, sexual therapy, and some other mixed treatments covering education, cognitive behavioral therapy, and mindfulness-based therapy.

#### Exercise and rehabilitation interventions (n = 2)

The numbers of these sessions were variable from 2-7 in a week, from 8-12 wk, and the time of each session was 30-90 min (47, 52). Most of the exercise and rehabilitation interventions focused on the genital and pelvic muscles training. All studies in this group had a positive effect on sexual dysfunction.

#### Medical interventions (n = 6)

In the studies with medical interventions on men (n = 4), Sildenafil were used in 2 studies and could improve erections, orgasmic function, sexual desire, satisfaction in sexual activity, and successful penetration (53, 54). Tadafil could improve erectile function, desire, orgasm, intercourse satisfaction, and successful penetration (55, 56). In the studies with medical interventions on women (n = 2), Sildenafil improved lubrication and sensation (57), and Onabotulinum/A reduced sexual dysfunction and improved all the FSFI subscales (58).

#### Multi-type interventions (n = 1)

One study was conducted based on pelvic floor muscle exercise and mindfulness, which did not significantly affect sexual dysfunction (59). Generally, most studies (n = 14) had improved at least a part of sexual dysfunction.

**Table 2:** Sexual function difference among groups (psych educational)


	**Pre-test **				**Post-test**			
**Study, year (Ref)**	Experimental*	Control*	**Weight (%)**	**Mean difference**	Experimental*	Control*	**Weight (%)**	**Mean difference**
	** Ghazagh, 2019 (20)**	12 (40.59 ± 12.34)	12 (36.15 ± 7.72)	46.4	4.44 [-3.80, 12.68]	12 (46.9 ± 8.28)	12 (35.1 ± 5.37)	66.0	11.80 [6.22, 17.38]
** Khodaveisi, 2018 (22)**	37 (60.9 ± 35.1)	37 (70.1 ± 31.2)	13.7	-9.20 [-24.33, 5.93]	37 (68.9 ± 27.7)	32 (67.3 ± 30.9)	10.6	1.60 [-12.34, 15.54]
** Hamidizadeh, 2009 (21)**	35 (61.48 ± 18.53)	35 (70.86 ± 34.12)	19.0	-9.38 [-22.24, 3.48]	35 (75.2 ± 11.36)	35 (50.15 ± 29.74)	0.0	25.05 [14.50, 35.60]
** Mirzaei, 2017 (24)**	10 (38 ± 15.58)	10 (46.5 ± 12.21)	20.9	-8.50 [-20.77, 3.77]	10 (43.9 ± 12.08)	10 (41.9 ± 9.1)	23.4	25.05 [14.50, 35.60]
** Total (95% CI)**	94 (-)	94 (-)	100.0	-2.76 [-8.37, 2.84]	94 (-)	94 (-)	100.0	2.00 [-7.37, 11.37]
** Heterogeneity**	Chi^2^: 5.49, df: 3, (p: 0.14), I^2^: 45%				Chi^2^: 4.13, df: 2, (p: 0.13), I^2^: 52%			
** Test for overall effect**	Z: 0.97 (p: 0.33)				Z: 3.64 (p: 0.0003)			
Data presented as number (Mean ± SD)								

**Table 3:** Sexual function difference among groups (exercise and rehabilitation)


	**Pre-test**				**Post-test**			
**Study, year (Ref)**	Experimental*	Control*	**Weight (%)**	**Mean difference**	Experimental*	Control*	**Weight (%)**	**Mean difference**
	** Ahadi, 2013 (26)**	10 (64.16 ± 34.49)	10 (81.67 ± 19.16)	5.0	-17.51 [-41.96, 6.94]	10 (73.33 ± 33.52)	10 (85.01 ± 17.9)	6.4	-11.68 [-35.23, 11.87]
** Ahadi, 2013 (26)**	11 (67.42 ± 34.45)	10 (81.67 ± 19.16)	5.4	-14.25 [-37.82, 9.32]	11 (75.75 ± 32.8)	10 (85.01 ± 17.9)	7.1	-9.26 [-31.59, 13.07]
** Ahmadi, 2010 (27)**	11 (67.42 ± 34.45)	11 (81.67 ± 19.16)	5.5	-14.25 [-37.55, 9.05]	11 (75.75 ± 32.8)	10 (85.01 ± 17.9)	7.1	-9.26 [-31.59, 13.07]
** Azimzadeh, 2013 (29)**	43 (95 ± 38.75)	24 (55.89 ± 39.52)	Not estimable		16 (100 ± 39.93)	18 (46.42 ± 46.9)	0.0	53.58 [24.39, 82.77]
** Kargarfard, 2012 (30) **	16 (44 ± 21.4)	16 (40.5 ± 16.3)	17.3	3.50 [-9.68, 16.68]	11 (47.6 ± 20.3)	10 (41.7 ± 11.8)	18.0	5.90 [-8.15, 19.95]
** Najafi Dolatabad, 2012 (36)**	30 (36.25 ± 38.4)	30 (27.5 ± 32.5)	9.3	8.75 [-9.25, 26.75]	30 (30 ± 32.37)	30 (27.5 ± 32.5)	13.2	2.50 [-13.91, 18.91]
** Romberg, 2005 (33)**	65 (67.2 ± 27.5)	58 (69.8 ± 18.7)	40.0	-2.60 [-11.26, 6.06]	47 (68.5 ± 29.7)	48 (75.9 ± 26.3)	27.9	-7.40 [-18.69, 3.89]
** Sutherland, 2001 (35)**	11 (61.87 ± 24.37)	11 (79.37 ± 16.87)	9.8	-17.50 [-35.02, 0.02]	11 (68.12 ± 23.75)	11 (66.87 ± 25)	8.6	1.25 [-19.13, 21.63]
** Sutherland, 2005 (34)**	11 (68.75 ± 22.5)	11 (54.37 ± 24.37)	7.8	14.38 [-5.22, 33.98]	11 (66.25 ± 21.25)	11 (48.12 ± 20.62)	11.6	18.13 [0.63, 35.63]
** Total (95% CI)**	156 (-)	157 (-)	100.0	-2.65 [-8.12, 33.98]	142 (-)	140 (-)	100.0	-0.53 [-6.49, 5.44]
** Heterogeneity**	Chi^2^: 11.34, df: 7, (p: 0.12), I^2^: 38%				Chi^2^: 8.79, df: 7, (p: 0.27), I^2^: 20%			
** Test for overall effect**	Z: 0.95, (p: 0.34)				Z: 0.17, (p: 0.86)			
*Data presented as number (Mean ± SD)								

**Table 4:** Sexual function difference among experimental and control groups in (medical)


	**Pre-test**				**Post-test**			
** Study, year (Ref)**	Experimental*	Control*	**Weight (%)**	**Mean difference**	Experimental*	Control*	**Weight (%)**	**Mean difference**
	** Simone, 2006 (39)**	41 (71.5 ± 27)	77 (83.1 ± 26.6)	61.6	-11.60 [-21.78, 1.42]	41 (69.5 ± 36.5)	77 (77.1 ± 35.6)	48.8	-7.60 [-21.31, 6.11]
** Nozari, 2019 (42)**	25 (65.98 ± 22.28)	25 (57.14 ± 25.93)	8.84	-1.84 [-14.73, 11.05]	25 (65.98 ± 22.28)	57.14 ± 25.93	8.84	-4.56 [-4.56, 22.24]
** Total (95% CI)**	66 (-)	102 (-)	100.0	-7.84 [-15.84, 0.14]	66 (-)	102 (-)	100.0	0.81 [-4.56, 22.24]
** Heterogeneity**	Chi^2^: 1.36, df: 1, (p: 0.24), I^2^: 26%				Chi^2^: 2.82, df: 1, (p: 0.09), I^2^: 65%			
** Test for overall effect**	Z: 1.93, (p: 0.05)				Z: 0.17, (p: 0.87)			
*Data presented as number (Mean ± SD)								

## 4. Discussion 

### About sexual function

As we noted among the evidence related to sexual function (n = 27), most articles (n = 23) were focused on promoting quality of life, which sexual function is one of its subscales. At the same time, only 4 studies focused on sexual function as the main variable. It is noteworthy that most of the interventions were not based on sexual issues specifically. They were not designed to improve sexual function, and the purpose of studies was to improve quality of life. Therefore, it is expected that the majority will not affect sexual function. Most studies in the psychoeducational category (5/7) significantly affected sexual functions or its subscales. Change in sexual function in MS sometimes results from psychological, emotional, social, and cultural aspects (9), so psychological and educational interventions are logical procedures for improving sexual function. As Chow et al. indicated in a systematic review, psychoeducational interventions appeared to benefit the sexual life of gynecological cancer women (60).

Among 13 studies in the exercise and rehabilitation category, just one of them could improve sexual function (2/12), the main variable of 11 articles in this category was quality of life, and most of the exercises were designed to enhance the quality of life not sexual function specifically. However, aerobic activity was effective on sexual function. As a study, aerobic exercise could be considered non-pharmacologic treatment with fewer side effects in increasing sexual desire in middle-aged women (61). However, 2 other articles were included in a current systematic review examined whether aerobic exercises did not have a positive effect on sexual function (28, 32).

Vitamin D addition to MS therapeutics drugs improved on sexual function (41), Vitamin D therapy could improve sexual function (62). However, folic acid and vitamin B12 did not have any significant effects. Medical interventions based on MS treatment did not positively affect on sexual function. A change in sexual function in MS can be related to many factors such as drug interventions and complications of symptoms treatment of the disease (9).

One of the multi-types interventions studies was based on all 3 interventions, improving sexual function in the second follow-up 2 yr later (44). This was a cohort study, and given the significant dropout rate at the second follow-up, the remaining participants likely were those who experienced the positive impact of the intervention. There were 7 studies conducted on only women, and all of them were in Iran. Otherwise, men did not participate specifically in any investigation. According to prevalence and incidence of MS in Iran (2019), the incidence of MS was estimated 16.5 in 100,000 men (95% CI: 13.7-23.4) and 44.8 in 100,000 women (95% CI: 36.3-61.6) (63). So Iranian women have a medium-to-high prevalence rate of MS, and maybe this is the cause of the researcher's attention to women with MS compared to men. Considering the positive effect of more than half of psychoeducational interventions and meta-analysis, which showed a significant improvement (p = 0.0003) of sexual function after interventions, these types seem to be more acceptable than others are in this category.

### About sexual dysfunction

15 articles were conducted to improve sexual dysfunction, in which the inclusion criteria for participants was the existence of sexual dysfunction. All of the included studies had specialized interventions in sexual issues accordingly all interventions had a positive effect on at least 2 subscales of sexual dysfunction, but just one of them had multi-types-interventions (mindfulness and Pelvic floor muscle exercise) didn't have a significant impact.

Among sexual dysfunction articles, all Psychoeducational interventions positively affected sexual dysfunction, and a systematic review, showed psychological interventions are effective treatment options for sexual dysfunction (64). Since 2 exercise and rehabilitation interventions focused on pelvic muscle training, they could have a positive effect on sexual dysfunction. A study showed a significant improvement of sexual function in the use of supervised pelvic floor muscle training (65). Only 4 articles were conducted on men's erection disorder (53-56), and all of them were in the category of medical interventions that used Sildenafil or Tadafil, and all could improve erectile function. Doggrell and colleagues found that Tadalafil is similarly effective as a Sildenafil in treating erectile dysfunction. It may be due to its longer duration of action (66).

Medical interventions for managing sexual dysfunction in women included Sildenafil which had a significant effect on lubrication and sensation and enhanced sexual desire, arousal, orgasm, and sexual satisfaction (67). Also, a single Onabot/A for treatment of overactive bladder could improve all subscales of FSFI. All these medications seem to be valid if it resolves organ with non-effective function, which involves sexual activity.

Interventions on men specifically include medical interventions that have been effective in the betterment of sexual dysfunction. This is probably because of a high prevalence of erectile dysfunction which 23-91% suffer from erectile dysfunction (68-71). This issue requires interventions with fast effects, and maybe this is the first step but not the only way of male sexual dysfunction treatment. It seems focused sex group therapy showed greater efficacy on erectile dysfunction in non-patient men. Also, men who received psychotherapy showed significant improvement in successful intercourse rate (72).

Due to the interaction between couples' sexual dysfunction and, on the other hand, the interference of MS with couples' life, this situation can affects sexual intimacy and marital relationship, we recommend that studies will design for couples.

### Strengths and limitations

This study had several strengths, a comprehensive literature search with no restrictions regarding genders (men or women) and questionnaires (specific or general scales with related subscale). In addition, for more information, this study included all interventional studies without limitations.

Due to the limited number of effective studies in each category and the no use of a similar questionnaire, it was impossible to use meta-analysis in all subgroups and compare the results for greater effectiveness among interventions. It was impossible to compare gender-based interventions exactly because the articles were inadequate, and in men, non-pharmacological methods were not specifically performed.

## 5. Conclusion 

Generally, psychoeducational and medical interventions with (10/12) 83.3% and (8/11) 72.7% respectively were the commonest types of effective interventions. More than half of the psychoeducational studies (7/12) conducted specifically on women had a positive impact. On the other hand, only 4 articles were specifically targeted at men; researchers appear to be more interested in examining psychoeducational interventions on women and medical interventions on men.

### Recommendations for future research

Future research should establish effective psychological interventions for sexual functions given that an overall lack of RCT studies characterizes this field. There is a lack of RCTs addressing the effect of pelvic muscle training on sexual dysfunction of men or female with MS, and more high-quality RCTs is warranted.

##  Conflict of Interest

The authors declare that they have no conflict of interest.

##  Funding

No financial support was received for this part of the research.
